# Inspiratory muscle training in children with neuromuscular disorders

**DOI:** 10.4102/sajp.v80i1.2055

**Published:** 2024-08-08

**Authors:** Anri Human, Lieselotte Corten, Eleonora Lozano-Ray, Brenda M. Morrow

**Affiliations:** 1 Department of Physiotherapy, School of Health Care Sciences, Sefako Makgatho Health Sciences University, Pretoria, South Africa; 2 Department of Health and Rehabilitation Sciences, Faculty of Health Sciences, University of Cape Town, Cape Town, South Africa; 3 Department of Physiotherapy, Faculty of Health Sciences, University of Pretoria, Pretoria, South Africa; 4 Department of Physiotherapy, School of Health Sciences, University of Brighton, Eastbourne, United Kingdom; 5 Department of Physiotherapy, Red Cross War Memorial Children’s Hospital, Cape Town, South Africa; 6 Department of Paediatrics and Child Health, Red Cross War Memorial Children’s Hospital, Cape Town, South Africa

**Keywords:** inspiratory muscle training, inspiratory muscle strength, neuromuscular disorders, adolescents, children

## Abstract

**Background:**

Progressive respiratory muscle weakness and ineffective cough contribute to morbidity and mortality in children with neuromuscular disorders (NMD). Inspiratory muscle training (IMT) aims to preserve or improve respiratory muscle strength and reduce respiratory morbidity. This study aimed to determine the safety and efficacy of IMT in children with NMD.

**Methods/design:**

A randomised cross-over study compared a 3-month intervention (IMT) with control periods (no IMT). Children diagnosed with NMD (5 years – 18 years) performed 30 breaths (at 30% of maximum inspiratory mouth pressure [Pimax]) with an electronic threshold device, twice daily. During the control period, participants did not perform any IMT.

**Discussion:**

Twenty three children (median [interquartile range {IQR}] age of 12.33 [10.03–14.17] years), mostly male (*n* = 20) and non-ambulant (*n* = 14) participated. No adverse events related to IMT were reported. No difference in median patient hospitalisation and respiratory tract infection (RTI) rates between non-training and intervention periods (*p* = 0.60; *p* = 0.21) was found. During IMT, Pimax and peak cough flow improved with a mean ± standard deviation (s.d.) of 14.57 ± 15.67 cmH_2_O and 32.27 ± 36.60 L/min, compared to 3.04 ± 11.93 cmH_2_O (*p* = 0.01) and −16.59 ± 48.29 L/min (*p* = 0.0005) during the non-training period. Similar to other studies, spirometry did not show a significant change.

**Conclusion:**

A 3-month IMT programme in children with NMD appears safe and well-tolerated, with significant improvement in respiratory muscle strength and cough efficacy.

**Clinical implications:**

Inspiratory muscle training could be considered a cost-effective adjunct to respiratory management in children with NMD.

**Trial Registration:**

Pan African Clinical Trial Registry, PACTR201506001171421, https://pactr.samrc.ac.za.

## Introduction

Progressive inspiratory muscle weakness is associated with severe respiratory complications in individuals with neuromuscular disorders (NMD) (Chiang, Mehta & Amin 2018; Morrow et al. 2019; Panitch 2017; Toussaint et al. 2018). Sequelae of respiratory muscle weakness include hypoventilation, secretion retention, airway obstruction, increased work of breathing, and recurrent lower respiratory tract infections (RTI) (Chiang et al. 2018; Dohna-Schwake et al. 2006; Panitch 2017; Toussaint et al. 2018).

Inspiratory muscle training (IMT) aims to improve inspiratory muscle strength, optimising ventilation and cough ability, potentially reducing respiratory morbidity and improving health-related quality of life (HRQoL) (Gozal & Thiriet 1999; Wenninger et al. 2019). Inspiratory muscle training appears to be safe for people with NMD, however results are variable (Human, Honey & Morrow 2019; Human & Morrow 2021; Wanke et al. 1994; Winkler et al. 2000). Despite the potential pulmonary function benefits, IMT has not yet been included in NMD clinical practice guidelines owing to insufficient evidence of efficacy and safety, particularly in patients with dystrophinopathies (Eagle 2002; Finder et al. 2004; Sander et al. 2000; Silva et al. 2019; Topin et al. 2002). Furthermore, there is no consensus regarding IMT devices, optimal dosage or frequency for the wide pathophysiological scope of NMD (Núñez, Araos & Delgado 2014; Wenninger et al. 2019). Although IMT might improve respiratory function and reduce pulmonary morbidity in patients with NMD, exercise could accelerate muscle fatigue and muscle damage due to decreased blood flow, increased inflammation, and the replacement of muscle fibres with fatty deposits and fibrosis. The risk is greater in patients with dystrophinopathies than in other NMD because of the underlying pathophysiology influencing the structural proteins of the muscle membrane and the possible lack of protective mechanism of nitric oxide release during exercise (Sander et al. 2000; Tidball & Wehling-Henricks 2014; Woszezenki, Paulo Heinzmann-Filho & Donadio 2017).

Adequately powered clinical trials of IMT among children with NMD are extremely limited, show contradictory findings, and lack external validity and generalisability. Therefore, randomised controlled trials (RCT) are recommended (Human et al. 2017, 2019; McCool & Rosen 2006). Using a threshold device at an intensity of 30% of maximum inspiratory mouth pressure (Pimax), twice a day for 6 weeks was safe, effective, well-tolerated and acceptable among South African children living with NMD (Human & Morrow 2021). Hence, this study aimed to determine if a 3-month IMT programme, as an adjunct treatment strategy, is safe and effective among children (5 years – 18 years) with NMD.

## Research methods and design

### Study design

**FIGURE 1:**
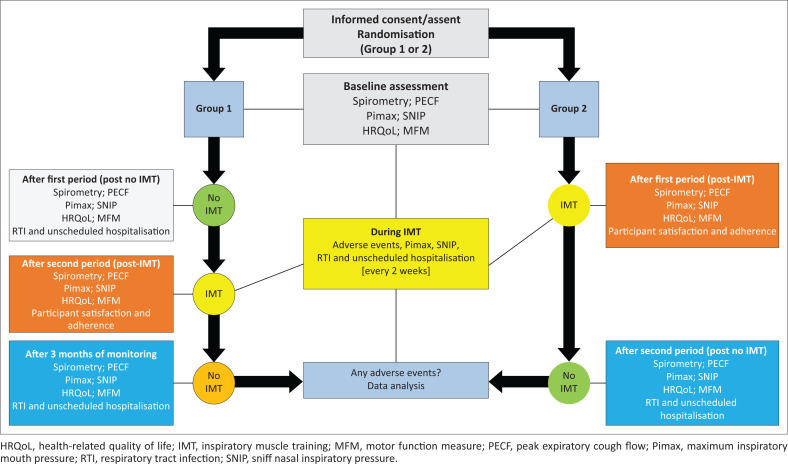
Group allocation for randomised controlled cross-over study and measurement points.

This randomised controlled, cross-over study randomly allocated consecutive participants to either Group 1 (first period control or non-training) or Group 2 (first period intervention), using a randomised opaque envelope system to ensure allocation concealment. Randomisation and allocation were conducted in each province separately, by an independent administrator at the academic hospital. The two groups and measurement points are indicated in Figure 1.

### Participants

Children and adolescents with a genetic and/or specialist confirmed diagnosis of NMD (5 years – 18 years), from two provinces in South Africa (Gauteng; Western Cape), at public hospitals, schools (public and private) and/or homes, were recruited over 2 years (2016–2018). Because of the rarity of NMD, and a lack of an electronic, centralised patient database and specialised NMD centres in South Africa at the time of the study, as compared to other countries, numerous recruitment sites had to be included in order to reach the calculated patient sample. Eligible children and adolescents were identified and recruited at the NMD clinics of two academic hospitals, or through referrals from healthcare professionals working in the field of NMD, the Muscular Dystrophy Foundation of South Africa, and patients and/or families already involved in the study. This study was performed as a single study process. The addition of study sites such as schools for children with special needs was included because of insufficient recruitment, as an approved amendment. Exclusion criteria were: vital capacity (VC) < 25% predicted, in the end of life period, concurrent acute respiratory tract infection (RTI) and/or fever, previous spontaneous pneumothorax, recent lung surgery, and participation in other clinical trials.

### Data collection tools and procedure

Primary outcome measures included any adverse events potentially related to IMT, the number of unscheduled hospitalisations for respiratory complications, and acute bacterial or viral respiratory infections during the intervention (IMT) and non-training periods.

Secondary outcome measures included functional ability (Brooke, Vignos and Motor Function Measure [MFM] scores), pulmonary function (spirometry, cough ability and inspiratory muscle strength), participant adherence to and satisfaction with the IMT protocol and HRQoL (participant satisfaction and HRQoL reported elsewhere). For this study, the primary and secondary outcome measures related to pulmonary function are described. Outcome measures were assessed at baseline and/or at pre-defined measuring points (Figure 1).

Pulmonary function assessment is commonly used to determine severity, functional impairment and disease progression among patients with muscle weakness. Vital capacity specifically is useful in evaluating respiratory status at diagnosis, in monitoring disease progression, indicating cough efficacy and predicting the risk of post-surgery complications and survival in patients with NMD (Dohna-Schwake et al. 2006; Chiang et al. 2018). Forced vital capacity (FVC) is a reliable general outcome measure for both inspiratory and expiratory muscle function and is indicative of general disease progression (Finder et al. 2004; Morrow et al. 2019). The feasibility of performing pulmonary function testing among young school children has been confirmed (Bianchi & Baiardi 2008; Nève et al. 2013).

Spirometry (VC, FVC, forced expiratory volume in one second [FEV_1_]; peak expiratory flow [PEF]) and cough ability (peak expiratory cough flow [PECF]) were assessed for all participants at baseline, after the first period, the second period and for those allocated to Group 1, 3 months post-monitoring (a third period) (Figure 1). These pulmonary function tests were performed in a relaxed upright sitting position (Winkler et al. 2000; Dohna-Schwake et al. 2006), based on standard practice as outlined in the American Thoracic Society/European Respiratory Society (ATS/ERS) guidelines (Miller et al. 2005). Spontaneous or relaxed (slow VC measured on expiration [EVC]) and forced spirometry (FVC, FEV_1_, PEF) were measured with a portable device (MicroLoop; Carefusion), whereas PECF was measured using a Paediatric Wright Peak Flow Meter with a mouthpiece (Chiang et al. 2018; Dohna-Schwake et al. 2006; Park et al. 2010).

Measures of inspiratory pressure might provide a better indication of survival rate and be a better predictor of respiratory insufficiency than spirometric measures of VC and FVC. A variety of respiratory muscle strength tests might be useful in NMD, as the patterns of respiratory muscle weakness differ depending on the type of NMD (Nève et al. 2013). Maximum static inspiratory pressure is one of the most sensitive indicators of decreased inspiratory muscle strength (Stefanutti et al. 2000). The most frequently used inspiratory muscle strength tests in children include Pimax and sniff nasal inspiratory pressure (SNIP) (Chiang et al. 2018). The SNIP is a reliable and valid alternative to Pimax which might be more natural and easier to perform, with a reduced learning effect and does not require practise or oral control (Fauroux & Aubertin 2007; Nève et al. 2013; Nicot et al. 2006).

Inspiratory muscle strength was measured in a sitting position using an electronic handheld device (MicroRPM; Carefusion) (Chiang et al. 2018; Dohna-Schwake et al. 2006; Morrow et al. 2019; Park et al. 2010). Maximum inspiratory mouth pressure (Pimax) was measured after full expiration (from residual volume), followed by a maximal inspiratory effort which was held for at least one second (Fauroux 2003; Fauroux & Aubertin 2007; Park et al. 2010). Participants also performed a sniff manoeuvre (SNIP) at the end of tidal volume (FRC) (Nève et al. 2013) with a nasal probe size that ensured complete closure of one nasal passage while the other nostril remained open to allow the passage of air (Stefanutti et al. 2000). At least three spirometry, PECF, Pimax and SNIP efforts were recorded within 20% of each other and the best value was used for analysis, as recommended (Fauroux & Aubertin 2007; Miller et al. 2005; Nicot et al. 2006). Inspiratory muscle strength (Pimax, SNIP) was measured at baseline, every 2 weeks for 3 months during IMT and then monthly during the non-training/monitoring period (Figure 1). The measurements were taken by the primary investigator and two research assistants in the various research settings. The primary investigator trained both research assistants in the use of the data collection forms and devices for baseline and outcome assessment. Baseline and follow-up assessments were performed by the researcher and research assistants either at the hospital, school or at participants’ homes in order to reduce the burden on families.

Inspiratory muscle training was performed for 30 breaths, at 30% of Pimax, with an electronic threshold device (Powerbreathe K3, HaB International Ltd, Southam, UK), twice daily, at school (5 days a week) or home (5 days – 7 days a week) for a duration of 3 months. The decision for training at home versus school was taken on an individual/preference basis, but in all cases IMT was performed. This study was a pragmatic (real-world) trial, with intention-to-treat analysis. Intervention frequency could not be limited in the experimental group, when training at home, but parents and/or caregivers and participants were informed that they were expected to perform IMT for at least 5 days a week. This programme was based on previous findings of a systematic review, pre-experimental study, and the manufacturer guidelines (Human et al. 2017; Human & Morrow 2021).

The IMT load was set according to the participant’s ability (Pimax) in order to be effective, but safe. To minimise the risk of muscle damage and improve participant adherence, an intensity of 30% of Pimax was implemented in this study (Topin et al. 2002; Woszezenki et al. 2017). The hypothesis was that this training intensity is the minimum intensity required for improved inspiratory muscle strength while not overexerting the patient (Hill et al. 2010; Lӧtters et al. 2002; Woszezenki et al. 2017).

Participants started their training with three sets of 10 breaths, twice a day, until they could perform 30 breaths consecutively. During the IMT period, training intensity (cmH_2_O) was adjusted based on Pimax values to ensure an effective training stimulus while not overexerting the participant and abiding to a training intensity of 30% of Pimax throughout the 3-month period. Adjustments were made every 2 weeks by the researcher and research assistants when they visited the participants at school or home to perform follow-up assessment and monitoring.

Performance and motivation were improved through visual feedback (counting down the breaths) provided on the screen of the Powerbreathe K3^®^ as well as an audible beep after each breath, with a double beep at the end of 30 breaths. Over and above the number of training sessions recorded on the Powerbreathe^®^ device, participants also kept a training diary, to monitor adherence to the IMT programme. Physiotherapists (at school), participants and/or parents and/or caregivers recorded daily outcome measures such as IMT repetitions, sets and level of perceived exertion (OMNI scale), adverse events and total number of training sessions in the provided training diaries. As part of safety and precautionary measures, in order to not overexert them, participants that trained during school hours were monitored by physiotherapists and those training at home were monitored by their parents and/or caregivers. The researcher and research assistants trained the school therapists and parents/caregivers on how to use the IMT device and monitor the participants, prior to commencement of the IMT programme. Participants were provided with a Powerbreathe K3 device for the duration of the training period, which was stored in a safe place either at school or home, depending on the participant’s preference.

During the 3-month control period, participants did not perform any IMT.

The level of perceived exertion was assessed with the OMNI pictorial scale (0–10 point visual analogue scale), recorded before and after every training session, as well as on average over every 2 weeks during the IMT period (Human et al. 2019; Pfeiffer et al. 2002; Utter et al. 2002).

The pulmonary function outcome measures and IMT protocol used in this RCT were previously shown suitable for the South African context and a NMD patient cohort (Human & Morrow 2021).

### Data management and analysis

Based on the mean Pimax difference observed in a previous pre-experimental study (Human & Morrow 2021), a sample of 24 patients was required to detect a minimum mean Pimax difference of 10 cmH_2_0 during the intervention period compared to no IMT (inactive control), over 3 months, with a power of 90% (1-beta) and 5% significance level (alpha). Therefore, 24 children (12 from Gauteng and 12 from Western Cape) were recruited, over 2 years.

Descriptive statistics were expressed as mean ± standard deviation (± s.d.) or median and interquartile range (IQR) for continuous variables, according to distribution. Frequency distributions (%) were provided where appropriate. To determine differences in continuous variables between IMT (experimental) and no IMT (control) periods, either a Student *t*-test for independent samples or a Mann–Whitney *U* test was performed depending on normality of distribution. Data were tested for normality using the Shapiro Wilks and Kolmogorov–Smirnov tests. The dependent Student *t*-test and non-parametric Wilcoxon matched pairs tests were used to test within group changes (comparing pre- and post-IMT variables). Change over time was analysed using repeated measures analysis of variance (ANOVA). Statistical significance was accepted as *p* < 0.05.

### Ethical considerations

Ethical approval and permission were provided by the University of Cape Town, Human Research Ethics Committee (HREC: 513/2015), provincial departments, schools and hospitals. Informed consent was obtained from parents or legal guardians of all participants, and assent was also obtained from the child participants. Although most participants understood English and Afrikaans, information leaflets and consent forms were available in Afrikaans, English, Xhosa and Setswana (commonly spoken languages in the two provinces). If needed, a translator was used during the interview, follow-up assessments and training on how to use the IMT device. This study is registered as a clinical trial (PACTR201506001171421).

## Results

### Bio-demographic description and functional ability of participants

**FIGURE 2:**
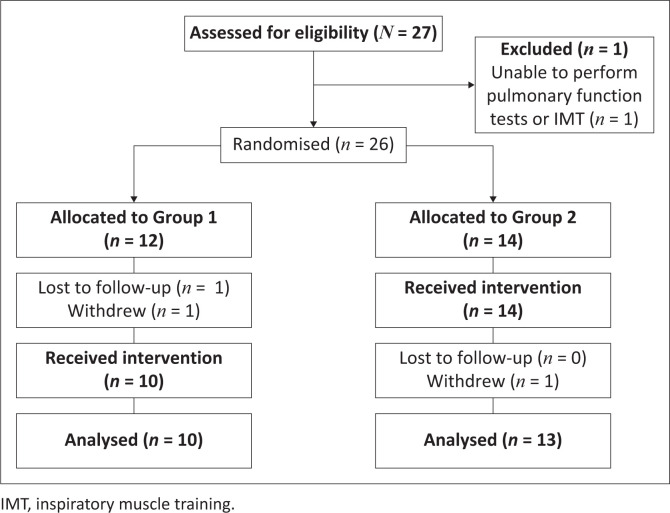
Patient recruitment and data collection process (CONSORT flow diagram).

A total of 23 participants (*n* = 20 males) with a median (IQR) age at enrolment of 12.33 (10.03–14.17) years and IMT naïve completed the trial (CONSORT framework: Figure 2).

The majority of participants had a confirmed diagnosis of Duchenne muscular dystrophy (DMD) (*n* = 13, 56.5%) and spinal muscular atrophy (*n* = 6; 26.1%) and were non-ambulant (*n* = 14, 60.9%) (Table 1). At recruitment, one participant with a tracheostomy received mechanical ventilation during the day and three participants used non-invasive ventilatory support at night (BiPAP). Another participant commenced with nocturnal BiPAP during the course of the study (post-IMT). The remaining 18 (78.3%) participants did not receive any ventilatory support during the study period.

**TABLE 1:** Bio-demographic data and functional ability of participants at baseline (*N* = 23).

Participant no.	Participant allocation	Sex (M/F)	Age (years)	NMD type	Weight-for-age (*Z*-scores)	BMI (*Z*-scores)	Mobility	Baseline (Brooke score/6)	Baseline (Vignos score/10)
1	G1	M	11.5	DMD	0.16	−0.76	A	1	3
2	G2	M	13.1	DMD	−1.92	−4.62	A	1	4
3	G1	M	13.3	DMD	−4.05	−10.27	NA	2	7
4	G2	M	15.9	DMD	−6.44	−15.45	NA	2	9
5	G1	M	15.9	DMD	−6.36	−16.53	NA	3	9
6	G2	M	8.7	DMD	0.65	−1.04	A	1	3
7	G2	M	12.4	CM	−1.45	−3.70	NA	4	9
8	G2	M	10.9	MD	−0.31	−0.60	A	1	2
9	G1	M	12.9	MD	0.16	0.00	A	1	3
10	G1	M	8.3	DMD	0.23	−4.20	A	1	2
11	G1	M	11.1	DMD	0.58	−0.10	NA	2	9
12	G2	M	12.3	DMD	−3.74	−1.80	A	1	2
13	G1	F	14.3	SMA II	−4.82	−5.37	NA	3	9
14	G1	M	11.1	DMD	1.44	0.56	NA	4	9
15	G2	M	10.1	DMD	0.70	−1.13	NA	1	8
16	G2	M	8.8	DMD	−0.73	−6.57	A	1	2
17	G2	F	14.3	SMA III	−3.90	−5.66	NA	2	9
18	G2	M	13.3	SMA III	−0.49	−1.37	NA	3	9
19	G1	F	6.7	SMA II	0.28	−6.24	NA	3	9
20	G1	M	6.1	DMD	−0.35	0.76	A	1	2
21	G2	M	14.2	SMA II	−4.61	−12.88	NA	6	9
22	G2	M	10.0	SMA II	−1.34	−7.62	NA	2	9
23	G2	M	16.4	CM	−4.50	−6.33	NA	3	9

A, ambulant; BMI, body mass index; CM, congenital myopathy; DMD, Duchenne muscular dystrophy; F, female; G1, group 1; G2, group 2; M, male; MD, muscular dystrophy; NA, non-ambulant; NMD, neuromuscular disorder; SMA, spinal muscular atrophy.

Seven participants (30.4%) reported co-morbidities such as gastro-oesophageal reflux (*n* = 1), cardiac pathology (*n* = 2) and other associated conditions such as autism spectrum disorder and/or attention deficit hyperactivity disorder (*n* = 4), but all could follow instructions. Eight participants (34.8%) also presented with spinal deformities (primarily scoliosis) at the time of intervention. At baseline, 10 participants (43.5%) were taking daily gluco-corticosteroids (mostly prednisone) as part of their chronic medication regime, five (21.7%) were taking cardiac function support medication, and six (26.1%) did not use any chronic medication at the time of recruitment. Two participants started taking corticosteroids during the intervention (IMT) period. A cardiologist cleared participants with cardiac pathology for enrolment into the trial.

Most participants used a wheelchair (manual or power or both) as reflected in the median (IQR) baseline recruitment Vignos score of 9 (3–9). Upper limb function was more preserved with baseline median (IQR) Brooke score of 2 (1–3), indicating the ability to raise arms above head only with flexed elbows.

Participants’ weight varied greatly with a median (IQR) of 30.00 (26.00–37.10) kg and a weight-for-age (WFA) *Z*-score of −0.73 (−4.05 to 0.23). Ten participants (43.5%) were within the normal body mass index (BMI) range, while 13 (56.5%) were severely underweight (Table 1). The median absolute BMI score was 12.70 (10.79–16.03) and *Z*-score was −4.20 (−6.57 to −0.76).

At recruitment, Groups 1 and 2 were well matched for bio-demographics (age, sex, type of NMD, weight and BMI) and pulmonary function (VC, FVC, FEV_1_; PEF; Pimax; SNIP & PECF), with no significant differences between the groups (Table 2).

**TABLE 2:** Bio-demographic and pulmonary function baseline data at recruitment based on group allocation (*N* = 23).

Variable	All participants (*N* = 23)	Group 1 (*n* = 10)	Group 2 (*n* = 13)	Allocation (*p*-value)
**Bio-demographic data**				
**Age (years)**	-	-	-	0.48
Median	12.33	11.29	12.42	-
IQR	10.03–14.17	8.25–13.25	10.08–14.17	-
**Sex**	-	-	-	0.81*
Male	20	8	12	-
Female	3	2	1	-
**Type of NMD**	-	-	-	0.44
DMD	13	7	6	-
SMA	6	2	4	-
CM	2	0	2	-
MD	2	1	1	-
**Weight (kg)**	-	-	-	0.93
Median	30.00	27.50	31.00	-
IQR	26.00–37.1	25.00–41.00	26.00–33.75	-
**WFA (Z-score)**	-	-	-	0.28
Median	−0.73	0.16	−1.45	-
IQR	−4.05 to 0.23	−4.05 to 0.28	−3.90 to -0.49	-
**BMI (kg/m^2^)**	-	-	-	0.41
Median	12.70	14.04	12.70	-
IQR	10.79–16.03	10.79–17.07	10.96–14.86	-
**BMI (Z-score)**	-	-	-	0.26
Median	−4.20	−2.48	−4.62	-
IQR	−6.57 to -0.76	−6.24 to 0.04	−6.57 to -1.37	-
**Pulmonary function**				
**VC (L)** †	-	-	-	0.16
Mean ± s.d.	1.38 ± 0.37	1.53 ± 0.26	1.28 ± 0.41	-
**FVC (L)**	-	-	-	0.63
Mean ± s.d.	1.59 ± 0.53	1.65 ± 0.48	1.54 ± 0.58	-
**FVC (Z-score)**	-	-	-	0.54
Mean ± s.d.	−3.47 ± 2.40	−3.11 ± 1.72	−3.75 ± 2.85	-
**FEV_1_ (L/s)**	-	-	-	0.57
Mean ± s.d.	1.38 ± 0.40	1.44 ± 0.33	1.34 ± 0.45	-
**FEV_1_ (Z-score)**	-	-	-	0.12
Mean ± s.d.	−3.34 ± 1.55	−2.77 ± 1.49	−3.78 ± 1.50	-
**PEF (L/min)**	-	-	-	0.66
Mean ± s.d.	189.13 ± 72.80	197.10 ± 51.00	183.00 ± 87.59	-
**PECF (L/min)** ‡	-	-	-	0.90
Mean ± s.d.	198.86 ± 98.83	195.56 ± 106.55	201.15 ± 97.51	-
**Pulmonary function (inspiratory muscle strength)**				
**Pimax (cmH_2_O)**	-	-	-	0.50
Mean ± s.d.	38.13 ± 22.65	41.90 ± 21.43	35.23 ± 23.98	-
**Pimax (% predicted value)**	-	-	-	0.51
Mean ± s.d.	56.47 ± 31.93	61.58 ± 27.16	52.55 ± 35.74	-
**SNIP (cmH_2_O)**	-	-	-	0.54
Mean ± s.d.	41.87 ± 21.43	45.10 ± 22.06	39.38 ± 1.48	-

Note: Bio-demographic data values are indicated as median (IQR); Pulmonary function values are expressed as mean (±s.d.).

BMI, body mass index; CM, congenital myopathy; DMD, Duchenne muscular dystrophy; FEV_1_, forced expiratory volume in one second; FVC, forced vital capacity; IQR, interquartile range; MD, muscular dystrophy; NMD, neuromuscular disorder; PECF, peak expiratory cough flow; PEF, peak expiratory flow; Pimax, maximum inspiratory mouth pressure; SMA, spinal muscular atrophy; SNIP, sniff nasal inspiratory pressure; VC, vital capacity; WFA, weight-for-age.

* , Yates *χ*^2^ *p*-value.

† , *n* = 19;

‡ , *n* = 22.

### Adverse events, respiratory tract infections, hospitalisations and perceived level of exertion

No serious adverse events related to IMT during the 3-month intervention period were reported.

Less than a third of participants (*n* = 7; 30.4%) reported nine RTI requiring antibiotics, during the study period (seven during the intervention and two during the non-training periods). Six episodes of hospitalisation in three participants occurred for respiratory-related conditions during the study period (five during the IMT and one during the non-training period). The majority of RTI (6/7; 85.7%) during the IMT period and all hospital admissions occurred during autumn and winter seasons, which is a high-risk season for respiratory infections. During acute RTI and hospitalisation, participants did not perform IMT as this is contra-indicated. There were however no significant differences in the median number of RTI or hospitalisation days per participant between the intervention (IMT) (median [IQR]: 0.00 [0.00–1.00]; 0.00 [0.00–0.00]) compared to control (non-training): 0.00 (0.00–0.00); 0.00 (0.00–0.00) periods (*p* = 0.60; *p* = 0.21).

The perceived levels of exertion were low during the first 2 weeks of IMT (median [IQR]: 3[2–4]), and reduced further (median [IQR]: 3 [1–4]) between intervention weeks 6 and 8 (*p* = 0.02). There was no statistically significant difference between the median OMNI score at 2 weeks of intervention (0–2 weeks) compared to the last 2 weeks of IMT (10 weeks – 12 weeks) (Wilcoxon *p* = 0.32). The difference in OMNI score (ΔOMNI) at the end of the intervention (post 3 months) and after the first 2 weeks of intervention indicated a decrease in perceived level of exertion (median [IQR] of −1.00 [−2.00 to 0.00]).

### Pulmonary function

Pulmonary function values (spirometry, PECF and inspiratory muscle strength) for the intervention or training period (IMT) and non-intervention or non-training period and differences between these groups are presented in Table 3.

**TABLE 3:** Pulmonary function during intervention inspiratory muscle training and non-training periods (*N* = 23).

Variable	IMT/intervention period (*N* = 23)				Non-training (control) period (*N* = 23)				Between-group difference in the change from baseline to 3 months	Between groups (*p*-value)
	Baseline	3 months	Difference	Within group (*p*-value)	Baseline	3 months	Difference	Within group (*p*-value)		
**VC (L)†**	-	-	-	0.63	-	-	-	0.17	-	0.20
Mean ± s.d.	1.42 ± 0.40	1.38 ± 0.38	−0.04 ± 0.32	-	1.41 ± 0.34	1.50 ± 0.45	0.09 ± 0.27	-	−0.12 ± 0.44	-
**FVC (L)**	-	-	-	0.49	-	-	-	0.90‡	-	0.53
Mean ± s.d.	1.55 ± 0.46	1.50 ± 0.43	−0.04 ± 0.31	-	1.59 ± 0.46	-	0.02 ± 0.41	-	−0.07 ± 0.63	-
Median	-	-	-	-	-	1.60	-	-	-	-
IQR	-	-	-	-	-	1.28–1.69	-	-	-	-
**FEV_1_ (L/s)**	-	-	-	0.44	-	-	-	0.09	-	0.27
Mean ± s.d.	1.34 ± 0.42	1.37 ± 0.37	-	-	1.43 ± 0.36	1.37 ± 0.38	-	-	-	-
Median	-	-	0.04	-	-	-	−0.05	-	0.06	-
IQR	-	-	−0.12 to 0.20	-	-	-	−0.11 to 0.02	-	−0.13 to 0.21	-
**PEF (L)**	-	-	-	0.94	-	-	-	0.51	-	0.76
Mean ± s.d.	189.09 ± 78.50	188.26 ± 64.71	−0.83 ± 51.49	-	200.09 ± 63.24	195.26 ± 67.00	−4.83 ± 34.36	-	4.00 ± 74.07	-
**Pimax (cmH_2_O)**	-	-	-	0.0002*	-	-	-	0.23	-	0.01*
Mean ± s.d.	40.57 ± 24.56	55.13 ±28.17	14.57 ± 15.67	-	46.61 ± 27.05	49.65 ± 24.66	3.04 ± 11.93	-	11.52 ± 23.62	-
**SNIP (cmH_2_O)**	-	-	-	−0.01*	-	-	-	0.17	-	0.45
Median	46.00	53.0	9.00	-	47.00	54.00	4.00	-	3.00	-
IQR	21.00–70.00	036.00–67.00	−2.00 to 21.00	-	38.00–66.00	33.00–75.00	−3.00 to 15.00‡	-	8.00 to 15.00	-
**PECF (L/min)**	-	-	-	0.0005*	-	-	-	0.12	-	0.0005*
Mean ± s.d.	200.87 ± 94.18	238.18 ± 96.12	32.27 ± 36.60	-	220.00 ± 96.08	204.57 ± 98.53	−16.59 ± 48.29	-	46.74 ± 67.36	-

Note: Values are expressed as mean (± s.d.) or median (IQR).

FEV_1_, forced expiratory volume in one second; FVC, forced vital capacity; IQR, interquartile range; PECF, peak expiratory cough flow; PEF, peak expiratory flow; Pimax, maximum inspiratory mouth pressure; SNIP, sniff nasal inspiratory pressure; VC, vital capacity; IMT, inspiratory muscle training.

* , statistically significant.

† , *n* = 19;

‡ , Wilcoxon rank test.

There was no significant change between the IMT and non-training periods for lung volumes and flows (VC, FVC, FEV_1_ and PEF) (Table 3). Mean (± s.d.) inspiratory muscle strength (Pimax) improved by 14.57 ± 15.67 cmH_2_O during the IMT period, compared to a 3.04 ± 11.93 cmH_2_O change during the non-training period (*p* = 0.01), but the change in SNIP was not significantly different between the two periods (*p* = 0.45). Mean (± s.d.) cough ability (PECF) increased during the training period by 32.27 ± 36.60 L/min), while the mean PECF declined by 16.59 ± 48.29 L/min during the non-training period (*p* = 0.0005). The improvements in Pimax, SNIP, and PECF pre-post IMT were maintained for 3 months following IMT, with no subsequent evidence of decline (Pimax *p* = 0.95; SNIP *p* = 0.14; PECF *p* = 0.17).

**FIGURE 3:**
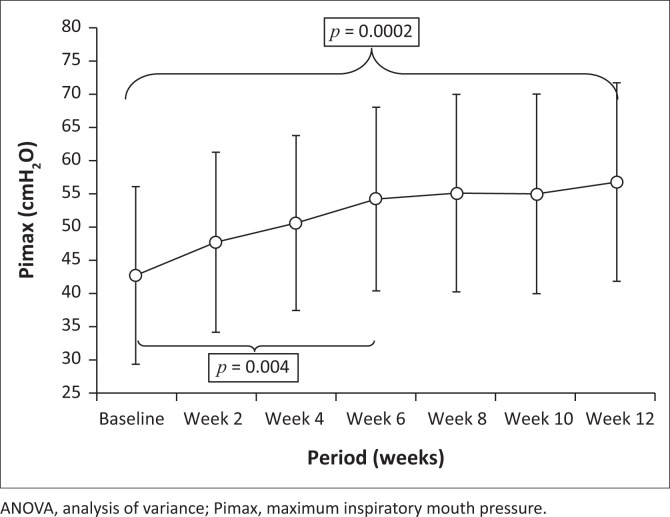
Change in inspiratory muscle strength (mean Pimax) values over the intervention period (ANOVA *p* = 0.00006). The vertical bars denote 0.95 confidence intervals. Significant post-hoc bivariate dependent tests between measurement points are indicated.

Within-group changes in Pimax and SNIP over the intervention period were statistically significant. Post-hoc *t*-tests for dependent variables showed significant increases between baseline and 6 weeks (Pimax *p* = 0.004; SNIP *p* = 0.03) and between baseline and 12 weeks (Pimax *p* = 0.0002; SNIP *p* = 0.01) (Figure 3).

### Inspiratory muscle training protocol adherence and participant satisfaction

Overall adherence to the IMT programme was good (range: 56.5% to 79.0%). The total number of training sessions for participants over the 3-month IMT period, as noted in their training diaries, ranged from 19 to 170 with a median (IQR) of 95 (64–159). A total of 120–168 training sessions were targeted and the number of training sessions recorded on the Powerbreathe^®^ devices (dependent on the quality of breaths) ranged from 10 to 169, with a mean (± s.d.) of 96.65 ± 48.45. The majority of participants (*n* = 19) had the same number of training sessions noted in their training diary as recorded on the Powerbreathe^®^ device, confirming the non-significant difference between the self-reported recordings and the device records (*p* = 0.43).

Overall participant satisfaction with the IMT programme on a 10-point visual analogue scale (VAS) was high, with a median (IQR) of 8 (5–10) reported by participants. The majority of participants (*n* = 18) and caregivers and/or parents (*n* = 20) also indicated that they would like to continue with IMT as treatment strategy, even after the study was completed.

## Discussion

This randomised cross-over trial showed IMT to be safe and effective in improving inspiratory muscle strength and cough ability with good patient adherence and high satisfaction levels in children with NMD, similar to a previous pre-experimental study (Human & Morrow 2021). There was no evidence of IMT causing either an increase in respiratory morbidity or a decrease in the number of infections or hospitalisations between intervention and non-training periods. Satisfactory levels of fatigue and exertion during training were reported.

### Participant characteristics

Participants (age, sex, weight, spinal deformity, type of NMD, functional ability and pulmonary function) were similar to those described in other studies of children with a variety of NMD (Jansen et al. 2013; LoMauro et al. 2014; Nève et al. 2013). The majority of participants were male, as expected, considering most presented with DMD, the most common type of NMD reported in the paediatric population (*n* = 13), especially among males (Gozal & Thiriet 1999; Koessler et al. 2001; Nicot et al. 2006; Winkler et al. 2000). Despite 60% (*n* = 14) of participants being non-ambulant at recruitment, which was expected as most were already in the pre- or adolescent phase (> 10 years), overall cardio-pulmonary function was fair, with the majority (*n* = 22) non-ventilator dependent, similar to other studies (LoMauro et al. 2014; Topin et al. 2002; Wanke et al. 1994; Yeldan, Gurses & Yuksel 2008).

Thirteen participants in this cross-over RCT were classified as severely underweight based on BMI *Z*-scores and the median BMI *Z*-score (−4.20) which is much lower than reported by Nève et al. (2013) among 33 children with DMD (−0.1 [−4.8 to −6.3]).

### Adverse events, hospitalisation, respiratory tract infections and perceived level of exertion

Similar to previous South African case and pre-experimental studies (Human et al. 2019; Human & Morrow 2021) and another study among children and adults with NMD (Winkler et al. 2000), this study reported no serious adverse events related to IMT.

Almost a third of participants (*n* = 7) experienced RTI over both the intervention (IMT) and non-training periods. There was no evidence of a difference in the number of RTI or hospitalisations per participant between the training and non-training periods (*p* = 0.21; *p* = 0.60). Most of the nine respiratory infections reported throughout the study occurred during the training period, which might be explained by the fact that six of these participants were performing IMT during the high-risk season for RTI (April to September).

The slight decrease in the OMNI score reported at 3 months (end of IMT intervention) as compared to the first 2 weeks of training was not significant (*p* = 0.32). Reasons for the lack of significant change could include: (1) the baseline values reported (after 2 weeks of IMT) were already relatively low with the majority reporting between 2 and 4 out of 10 which indicates ‘a little tired’ to ‘getting more tired’; (2) the OMNI scale might not be sensitive enough to detect small changes in perceived level of exertion; (3) could be affected by respiratory complications or illness; and (4) some children may have had difficulty understanding the abstract concept of exertion level (Pfeiffer et al. 2002). The lack of fluctuation in level of perceived exertion throughout the IMT period could also indicate appropriate levels of training intensity and adapting to the individual’s inspiratory muscle strength, reducing the risk of fatigue, overexertion and muscle damage.

### Pulmonary function

Most participants in this trial were in their pulmonary function decline phase (10 years – 18 years), non-ambulant and with PEF < 80% predicted. Similar to other studies (Winkler et al. 2000; Yeldan et al. 2008), no significant changes in spirometry (VC, FVC, FEV_1_ and PEF) were observed pre- to post-intervention nor was there any evidence of a difference between the intervention and non-training periods. This may be partly explained by the sigmoid shape of the pressure-volume curve, implying that improvements in inspiratory muscle strength (Pimax, SNIP) may not translate into increased lung volumes (Aboussouan 2009).

This study found a significant improvement in Pimax during the IMT period (14.57 ± 15.67 cmH_2_O) compared to the non-training period (3.04 ± 11.93 cmH_2_O) (*p* = 0.01). This is contrary to the findings by Martin et al. (1986), but similar to that of Gozal and Thiriet (1999) in 21 children with NMD (DMD and SMA III), which reported a similar difference between mean ΔPimax in the IMT (19.8 ± 3.8) versus the non-training group (4.2 ± 3.6; *p* = 0.02). Other studies have shown similar increases in Pimax associated with IMT (Human & Morrow 2021; Winkler et al. 2000).

The IMT and Pimax measurements in this study were both performed at residual volume, implying that measurement and training were of the same motor unit. Due to specific training and continued improvement over the 3 months of IMT, true strengthening of inspiratory muscles may have occurred and the effects seen may not solely be because of a learning effect (LoMauro et al. 2014; Silva et al. 2019). This could also explain why SNIP values did not improve significantly compared to the non-training period, as measurements for SNIP are taken at functional residual capacity. The SNIP values did, however, show a significant within group improvement over the 3-month intervention period (*p* = 0.01). The increased Pimax was maintained for 3 months after participants stopped IMT (*p* = 0.95) which is contrary to some previous reports (Eagle 2002; Martin et al. 1986), whilst other studies have also reported maintenance of improved inspiratory muscle strength after training cessation (Wanke et al. 1994; Wenninger et al. 2019). A previous experimental, long-term study (24 months) showed that IMT was associated with improved inspiratory muscle strength and endurance (Pimax; 12sMVV), supporting IMT use in children and adolescent with DMD and SMA (Koessler et al. 2001).

A correlation between PECF and lung volumes has been shown in children four to 18 years of age (Bianchi & Baiardi 2008; Gogou et al. 2017). This cross-over study showed a highly significant difference (*p* = 0.0005) between the mean (± s.d.) difference in PECF during IMT (32.27 ± 36.60L/min) and the non-training period (−16.59 ± 48.29L/min). These findings support the theory that improved inspiratory muscle strength translates into improved cough ability. The improved PECF after implementing the 3-month IMT programme could be explained by the increased ability to inhale sufficient volumes during the inspiratory phase of the cough (pre-cough volume), because of improved inspiratory muscle strength (Bianchi & Baiardi 2008; Gogou et al. 2017; McCool & Rosen 2006). From these and previous results, it appears that even patients with progressed disease, non-ambulant and/or with decreased oral control and/or ventilated can perform and may benefit from IMT to improve inspiratory muscle strength and cough efficacy with potential symptomatic relief (Human et al. 2019).

In this study, participants were very motivated and showed good adherence throughout the intervention period. The number of training sessions recorded in the training diaries and on the Powerbreathe^®^ devices were highly correlated. The overall good participant adherence in this cross-over RCT is similar to other studies conducted among children with NMD (Jansen et al. 2013; Wanke et al. 1994; Yeldan et al. 2008) and adults with late-onset Pompe disease (Wenninger et al. 2016). This could also be attributed to the high reported level of participant satisfaction with IMT (median: 8/10) as well as the generally positive attitude of the parents and/or caregivers and physiotherapists involved.

### Limitations of the study and research recommendations

Despite the relatively small sample size of 23 participants, this study is comparable to other international respiratory muscle training studies in children with NMD, favouring IMT, and supported by sample size calculation (Gozal & Thiriet 1999; Koessler et al. 2001; Martin et al. 1986; Yeldan et al. 2008). Seventeen of the participants presented with a muscle pathology (DMD, MD and CM) and six with SMA. The variation of conditions might affect the outcome of IMT; however, the common denominator is underlying respiratory muscle weakness, despite the pathophysiology. The risk for overexertion and adverse events such as muscle fibre breakdown, inflammation, and further muscle weakening because of increased fatty replacement of muscle fibres might however be higher in dystrophinopathies. Through monitoring perceived level of exertion (OMNI scale), the risk for adverse events such as fatigue, overexertion and muscle damage was reduced. The low levels of perceived exertion (2–4/10) throughout the training period, together with clinically relevant improvement may indicate that a training intensity of 30% Pimax can be safe and effective, even in dystrophinopathies. Future larger RCT should consider including patients with similar pathophysiology, such as dystrophinopathy only, in order to obtain condition-specific results.

Similar to other clinical trials among children with NMD (Jansen et al. 2013), the assessors were not blinded to group allocation, as this was not feasible. The researcher and research assistants had to teach participants how to perform IMT, monitor their level of exertion and adjust their training programme accordingly. The lack of blinding of assessors to allocation could however have introduced bias. Data were collected by three different individuals, which may constitute a risk of bias relating to inter-rater inconsistencies. Confirmation of inter-rater reliability is recommended for future studies.

Inspiratory muscle training might have a positive and clinically relevant effect for children with a wide variety of NMD, however, considering the majority of participants in this study had DMD, this may skew the results of this study and limit external validity. As NMD are chronic conditions, larger, longer-term and multisite clinical trials are required to determine the effect of IMT on clinical outcome measures, but also respiratory morbidity, HRQoL and patient experience (Gozal & Thiriet 1999; McCool & Rosen 2006; Wenninger et al. 2019).

## Conclusion

The findings of this cross-over RCT support the use of IMT as a readily available and inexpensive option, which may mitigate the problems associated with inspiratory muscle weakness and poor cough ability which are evident in this patient cohort.

This study did not identify evidence of a difference in the number of hospitalisations and respiratory infections requiring antibiotics between IMT intervention and non-training periods (suggesting non-inferiority compared to no treatment); there were no reported adverse events, and participant satisfaction and adherence were acceptable to high. Inspiratory muscle strength (Pimax) and cough ability (PECF) showed a significant improvement during the intervention compared to non-training periods, which has clinical relevance for children with NMD. Like other studies, no significant changes were observed between or within groups for spirometry.

### Clinical implications

The use of IMT therefore appears to be safe, well-tolerated and effective in improving inspiratory muscle strength and cough ability in children with a variety of NMD, including dystrophinopathies, and could be considered as an adjunct to respiratory management. Larger, longer-term RCT within different contexts are however warranted.
